# Seamless Micro-Electro-Mechanical System-Inertial Navigation System/Polarization Compass Navigation Method with Data and Model Dual-Driven Approach

**DOI:** 10.3390/mi15020237

**Published:** 2024-02-02

**Authors:** Huijun Zhao, Chong Shen, Huiliang Cao, Xuemei Chen, Chenguang Wang, Haoqian Huang, Jie Li

**Affiliations:** 1 The State Key Laboratory of Dynamic Measurement Technology, The School of Instrument and Electronics, North University of China, Taiyuan 030051, China; 2 The Intelligent Vehicle Research Center, School of Mechanical Engineering, Beijing Institute of Technology, Beijing 100081, China; 3 The College of Energy and Electrical Engineering, Hohai University, Nanjing 211100, China

**Keywords:** cubature Kalman filter, integrated navigation system, NARX, variational Bayesian method

## Abstract

The integration of micro-electro-mechanical system–inertial navigation systems (MEMS-INSs) with other autonomous navigation sensors, such as polarization compasses (PCs) and geomagnetic compasses, has been widely used to improve the navigation accuracy and reliability of vehicles in Internet of Things (IoT) applications. However, a MEMS-INS/PC integrated navigation system suffers from cumulative errors and time-varying measurement noise covariance in unknown, complex occlusion, and dynamic environments. To overcome these problems and improve the integrated navigation system’s performance, a dual data- and model-driven MEMS-INS/PC seamless navigation method is proposed. This system uses a nonlinear autoregressive neural network (NARX) based on the Gauss–Newton Bayesian regularization training algorithm to model the relationship between the MEMS-INS outputs composed of the specific force and angular velocity data and the PC heading’s angular increment, and to fit the integrated navigation system’s dynamic characteristics, thus realizing data-driven operation. In the model-driven part, a nonlinear MEMS-INS/PC loosely coupled navigation model is established, the variational Bayesian method is used to estimate the time-varying measurement noise covariance, and the cubature Kalman filter method is then used to solve the nonlinear problem in the model. The robustness and effectiveness of the proposed method are verified experimentally. The experimental results show that the proposed method can provide high-precision heading information stably in complex, occluded, and dynamic environments.

## 1. Introduction

Currently, global navigation satellite systems (GNSSs) and inertial navigation systems (INSs) are the most typical and widely used navigation methods in the field of the Internet of Things (IoT), such as the Internet of Vehicles (IoV) [1,2,3,4]. However, as a result of the rapid developments in bionic technology [5], some new types of bionic autonomous navigation technologies have emerged that are based on biological principles and can help unmanned platforms including unmanned aerial vehicles (UAVs) [6], unmanned vehicles (UVs) [7,8,9], and autonomous underwater vehicles (AUVs) to realize autonomous navigation. In recent years, polarization navigation based on atmospheric polarized light [10] has been noted as a more mature technique for bionic autonomous navigation technology. This technique has the advantages of zero cumulative error, high accuracy, and zero electromagnetic interference. However, when the sky region is occluded, the polarization information will be subject to interference, and the accuracy of the polarization compass (PC) will be affected. At this time, the common INS and the PC can be combined to form an integrated navigation system to improve the overall orientation accuracy. However, because of the rapid accumulation of INS errors that occurs over time [11,12,13,14], the navigation accuracy will continue to decline when this integrated navigation system is located in an occluded and dynamic environment for long periods. Therefore, it is necessary to explore a new integrated navigation method that can adapt to a dynamic environment when the polarization signal is lost completely.

The current solutions available to improve the accuracy of an integrated navigation system composed of an INS and a PC can be divided into two types. The first is intended to increase the robustness of the integrated navigation system by adding other sensor types, including global positioning system (GPS) sensors, magnetometers, odometers, star sensors, celestial body sensors, and binocular stereo cameras, among others, but this leads to increases in hardware costs, system power consumption, and system volume. The other solution is to improve the navigation accuracy by improving the integrated navigation model. For example, in [15], a polarization-based tight coupling model (PTCM) was established and a reliable fusion strategy was proposed to extract information from the PC and the INS. To solve the problem of attitude and heading determination for the polarization-based attitude and heading reference system (PAHRS), the system measurement model coupled the attitude and heading cumulative error of the INS closely [16]. Using the new polarization measurement error equation and the INS error equation, the INS/PC integrated navigation error equation was then established [17], and an autonomous and fast initial alignment was realized. In addition, to improve the heading angle accuracy, the system error source was analyzed [18], and a new calibration model was established on this basis to compensate for the installation error of PAHRS. To compensate for the longitude and latitude errors of the INS [19], a bionic positioning system model that combined the PC and the INS was established. A mathematical model of the rapid transfer alignment (RTA) with disturbance was established [20] and the grid heading solution for polarized light navigation was extended to the measurement process, which solved the low-quality attitude reference problem of the master INS.

The integrated navigation system’s accuracy can be improved substantially using the method above, but when the carrier moves in a complex occluded environment, the PC’s polarization information is lost completely; the method described above will then be unable to estimate the navigation information accurately, and it will also be unable to output high-precision navigation information continuously. The limitations of use of a single Kalman filter have motivated researchers to explore new methods to enhance the accuracy of integrated navigation systems during partial sensor navigation information interruptions. Due to the self-learning and fitting capabilities of neural networks, the precision of integrated navigation system can be improved by combining them with a Kalman filter. In [21], an adaptive neural fuzzy inference system algorithm based on variational Bayesian (VB) Kalman filtering and principal component analysis was proposed to prevent degradation of the navigation system’s positioning accuracy being caused by erroneous compensation. A Kalman fusion algorithm based on a backpropagation neural network (BPNN) was proposed [22], which used the current and past two-step information as inputs to the BPNN model; a relationship model between the INS velocity, the inertial measurement unit (IMU) output, the GPS interruption time, and the GPS position increment was then established to improve the integrated navigation system’s performance. By combining a Kalman filter with an improved multilayer perceptron network, a new hybrid fusion algorithm proposed in [23] provided pseudo-position information to assist the integrated navigation system. Because of the simple structure of the radial basis function (RBF) neural network, it is suitable for use in fast online training [24]. Various forms of hybrid prediction methods based on RBF neural networks and Kalman filters were proposed in [25], which improve the robustness of the integrated navigation system during the interruption of navigation information from partial sensors. To overcome the problem of increased navigation positioning errors caused by data interruption, ref. [26] developed a maximum correlation Kalman filter (KF) (mcKF) assisted by a dual free-size least-squares support vector machine (fLS-SVM) for fusing INS and UWB data. A finite impulse response (FIR) filter that combined predictive models and extreme learning machines (ELMs) was proposed [27] to improve the accuracy of the quadcopter positioning based on the UWB.

However, most of the combinatorial methods above are based on static neural networks, e.g., BPNN and RBF neural networks. Some methods only use the current and past two-step information as inputs, which cannot fit the dynamic characteristics of the integrated navigation system fully, and thus there is still room for further navigation accuracy improvement. With further extension of the research field, researchers have found that dynamic neural networks with strong learning abilities, memory retention abilities, and robustness, including the nonlinear autoregressive neural network (NARX), the long short-term memory (LSTM), and the gated recurrent unit (GRU) network, can make reasonable decisions based on the current input and historical information. These networks are also suitable for fitting and prediction of the time series. By considering the error accumulation of GRU network prediction, a hybrid algorithm based on the GRU and an adaptive Kalman filter was proposed in [28] to improve the navigation performance. To improve the position and velocity accuracy of the navigation system during GNSS outages, a new method was proposed in [29] that combined an unscented Kalman filter with the NARX, and the performance of the proposed method was validated experimentally using a real-world dataset.

The neural-network-aided navigation systems above have shown impressive performances. However, some neural network algorithms require large quantities of data and considerable computing resources to train effectively, and the predicted values inevitably contain errors. The disadvantages of the former case can be partially resolved by using appropriate data-driven models, while the disadvantages of the latter case can be suppressed by establishing appropriate error models. Therefore, this article proposes a dual data- and model-driven micro-electro-mechanical system–inertial navigation system (MEMS-INS)/PC seamless navigation method. In this method, when the PC signal is lost, the NARX is used to predict the heading angle increment and the VB cubature Kalman filter (VBCKF) algorithm is used to estimate the measurement noise covariance to improve the integrated navigation accuracy. The main contributions of this article are as follows:

1. The MEMS-INS/PC seamless navigation method driven by data and modeling is applied to the nonlinear MEMS-INS/PC integrated navigation system to provide a continuous and accurate navigation scheme.
2. A Gaussian–Newton Bayesian regularization algorithm, which is used to train the NARX, improves the accuracy and efficiency of the model significantly and increases the model’s stability. A nonlinear relationship between the angular rate and the specific force information of the INS and the PC heading angle increment is established by the NARX.
3. By considering the prediction error of the neural network, this study proposes a VBCKF algorithm with an inverse gamma distribution, introduces the variational Bayesian theory to estimate the variational measurement noise covariance, and improves the Kalman filter’s estimation accuracy in the presence of unknown measurement noise covariance and measurement outliers.

The rest of this article is organized as follows. Section 2 describes the integrated navigation system model and the fundamentals of the proposed algorithm in detail. The proposed dual data- and model-driven approach for seamless MEMS-INS/PC navigation is then presented in Section 3. In Section 4, field test results are given and the performance of the proposed algorithm is analyzed. Conclusions are finally presented in Section 5.

## 2. Integrated Navigation Model and Basic Algorithm

### 2.1. MEMS-INS/PC Loosely Coupled Navigation Strategy

The MEMS-INS/PC integrated navigation system mainly comprises the INS and the PC. The INS is composed of a three-axis gyroscope and an accelerometer that can measure the angular velocity and the linear velocity of the carrier. The PC is installed on top of the carrier and the carrier heading angle is calculated by obtaining atmospheric polarization information. 

**Figure 1:**
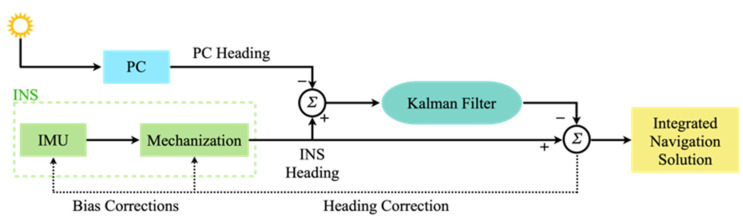
Block diagram of the loosely coupled MEMS-INS/PC system integration.

Since loosely coupled integration has the characteristics of small computation and easy realization [30], this paper integrates the INS and PC in a loosely coupled way (as shown in Figure 1). The INS provides continuous attitude information, and the PC can provide the vector heading information. The Kalman filter uses the difference between the INS and PC heading angle measurements to estimate the INS heading angle error and then feeds the error back to INS for correction. Because the method in this article only estimates the heading angle information optimally, the attitude error $\phi^{n}=\left[\begin{array}{c} \phi_{x}\;\phi_{y}\;\phi_{z} \end{array}\right]$, the three-axis gyro deviation $\varepsilon^{n}=\left[\begin{array}{c} \varepsilon_{x}\;\varepsilon_{y}\;\varepsilon_{z} \end{array}\right]$, and the accelerometer deviation $\nabla^{n}=\left[\begin{array}{c} \nabla_{x}\nabla_{y}\nabla_{z} \end{array}\right]$ are selected as the state ***X*** of the integrated navigation model:

(1) $$X={\left[\phi_{x}\;\phi_{y}{\;\phi }_{z}\;\varepsilon_{x}\;\varepsilon_{y}\;\varepsilon_{z}\nabla_{x}\nabla_{y}\nabla_{z}\right]}^{T}.$$

The error state equation of the integrated navigation model can be given as

(2) $$\dot{X}=FX+W,$$

where F is the state transition matrix; a detailed description of this matrix can be found in [31]. $W=\left[\begin{array}{c} {0_{1\times 3}\omega }_{g}\;\;\omega_{a} \end{array}\right]$ is the system’s Gaussian white noise, $\omega_{g}$ is the gyroscope noise, and $\omega_{a}$ is the accelerometer noise.

The measurement equation for the integrated model can then be given as

(3) Z=HX+V,

where $Z={\left[0_{1\times 2}{(\varphi }_{PC}-\varphi_{INS})0_{1\times 6}\right]}^{T}$ is the measurement quantity; $\varphi_{PC}$ is the heading angle measured by the PC, and $\varphi_{INS}$ is the heading angle measured by the INS; H is the measurement matrix, the specific derivation of which can be found by referring to [32]; and V is the measurement noise.

The integrated navigation system has strong nonlinear characteristics, and thus it is necessary to discretize the state equation and the measurement equation of the integrated navigation model as follows:

(4) $$\left\{\begin{array}{c} x_{k}=f\left(x_{k-1}\right)+\omega_{k-1}\\ z_{k}=h\left(x_{k}\right)+v_{k}\;\;\;\;\;\;\;\;\; \end{array}\right.\;$$

where $x_{k}$ is the state at time k; $z_{k}$ is the measurement at time k; $f\left(\cdot \right)$ and $h\left(\cdot \right)$ represent the state transition matrix F and the measurement matrix H after discretization, respectively; and $\omega_{k-1}\sim N(0,Q_{k-1})$ and $v_{k}\sim N(0,R_{k})$ are the mutually independent Gaussian noise. The discretized integrated navigation model of (4) is the essential part of the cubature Kalman filter (CKF), and the specific filtering process will be given in Section 3.

### 2.2. The NARX Neural Network

Unlike static neural networks, the NARX [33] is a nonlinear autoregressive model with external inputs that has feedback connections to capture the dynamic properties of time series data. The NARX can thus handle nonlinear time series data with autoregressive and external inputs, and it can learn the relationships between the inputs and the outputs of complex nonlinear dynamic systems. The output result from the NARX depends on the external input and the historical input and output at the current time. After nonlinear function processing, the network structure contains delay and feedback components, which enhance the adaptability of the network to time-varying laws, and the network also has the memory and correlation functions of the historical information. Therefore, the NARX offers great advantages in time-series prediction applications. In the integrated navigation system proposed in this article, the NARX can provide the predicted value of the PC heading angle increment based on the angular rate and specific force information output by the INS, which is essential to improve both the accuracy and the stability of the integrated navigation system. The mathematical expression for the NARX is given as

(5) $$\begin{array}{ll} y\left(t\right)= & g\left(u\left(t\right),u\left(t-1\right),u\left(t-2\right)\ldots ,u\left(t-n_{u}\right),\right.\\ & \left.y(t-1),y(t-2)\ldots ,y(t-n_{y}))\right. \end{array}$$

where y(t) represents the output of the neural network at time *t*, $g\left(\cdot \right)$ represents the nonlinear function obtained by neural network fitting, u(t) represents the input of the neural network, and $n_{u}$ and $n_{y}$ represent the maximum orders of the input and output delay, respectively.

**Figure 2:**
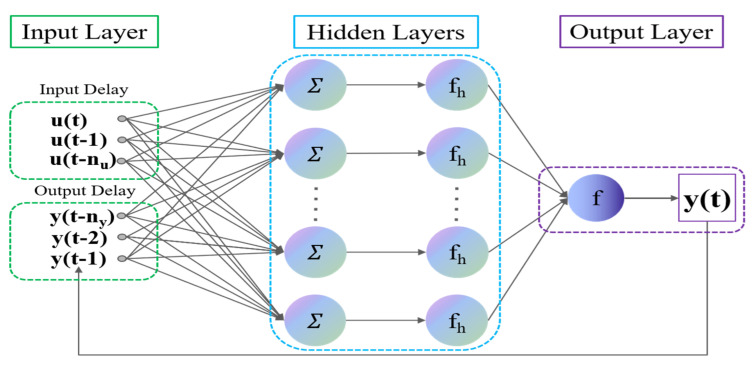
Architecture of the NARX.

The NARX model includes an input layer, hidden layers, and an output layer. Unlike a traditional neural network, the NARX input layer has a feedback connection that can provide the network output as an input to the network. Additionally, the input layer also contains external inputs and time series data. The number of nodes in the input layer is the same as the number of input values. The hidden layers are used to extract the nonlinear feature information from the data and then output this information to the output layer through a linear transformation to produce the final prediction results. The network performance can be improved by reasonable setting of the input delay, the output delay, and the numbers of hidden layers and nodes. Figure 2 shows the architecture of the NARX.

To obtain the optimal dynamic performance for the NARX, optimal dynamic adjustment of the network weights must be accomplished through training. Currently, most neural networks are trained using the Levenberg–Marquardt (LM) training algorithm, but when faced with more complex systems, such as limited datasets or complex model architectures, its generalization ability is poor, leading to poor network model accuracy. The Bayesian regularization algorithm, however, uses Bayesian principles to optimize the regularization parameters and improve the neural network’s generalization ability by correcting the neural network performance function. Therefore, this article uses the Gauss–Newton Bayesian regularization (GNBR) algorithm based on the Bayesian probabilistic model for training.

The purpose of a conventional neural network training algorithm is to reduce the mean squared error (MSE) $E_{D}$ of the neural network output:

(6) $$E_{D}=\frac{1}{N}\sum_{i=1}^{N}\;{\left(Y_{i}-y_{i}\right)}^{2}$$

where *N* is the number of samples, $Y_{i}$ is the target output, $y_{i}$ is the actual output, and the regularization method involves addition of a penalty term $E_{W}$ to $E_{D}$ for correction. The regularized network performance objective function can be expressed as

(7) $$E=\alpha E_{D}+\beta E_{W}$$

where $E_{W}=\frac{1}{M}\sum_{j=1}^{M}\;W_{j}^{2}$ is the sum of the squares of the network weights, α and β are the target parameters, *M* is the total number of weights, and $W_{j}$ represents the neural network connection weights.

Determination of the appropriate target parameters represents the main challenge after regularization, and the Bayesian regularization method can adjust the network weights according to the LM optimization theory, adjust the sizes of the target parameters α and β adaptively during the training process, and ensure these parameters are optimal. In the Bayesian framework, the neural network weights are regarded as random variables, and both these weights and the prior probabilities of the training samples are considered to follow Gaussian distributions. The optimal target parameters α and β can be solved based on the principle of maximizing the posterior probability:

(8) $$\alpha =\frac{M-\gamma }{2E_{D}},\beta =\frac{\gamma }{2E_{W}}$$

where γ is the number of effective weight values, and

(9) $$\gamma =N-2\beta tr\left(G^{-1}\right)$$

where G is the Hessian matrix of the objective function, which can be approximated by using the Gaussian–Newton method:

(10) $$G=\alpha \nabla^{2}E_{D}+\beta \nabla^{2}E_{W}.$$

Based on combination of the Hessian matrix approximated by using the Gaussian–Newton method with Bayesian regularization [34], the specific steps for the GNBR training algorithm are given in Algorithm 1.
**Algorithm 1:** GNBR Training Algorithm**Step 1:** Initialize the target parameters α, β$,\;\mathrm{and}\;W_{j}$.**Step 2:** Use the one-step LM algorithm to minimize the target network performance scale function E.**Step 3:** Approximate G by means of the Gaussian–Newton method and calculate the number of effective weights γ.**Step 4:** Update target parameters α and β with (8).**Step 5:** Repeat Steps 2–4 until the values of α and β converge.

## 3. Seamless MEMS-INS/PC Loosely Coupled Navigation Based on NARX-VBCKF

**Figure 3:**
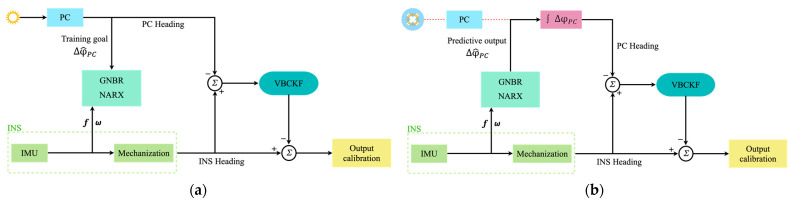
The working framework of the integrated navigation system. (**a**) Training model. (**b**) Prediction model.

In practical navigation applications, when the carrier moves in a complex occlusion environment, the heading angle information output by the PC will be unstable because of occlusion. At this time, optimal estimation cannot be achieved using the traditional single Kalman filter. The proposed dual data- and model-driven MEMS-INS/PC seamless integrated navigation system architecture includes two parts: the data-driven part and the model-driven part. The core of the data-driven part is the NARX based on GNBR, which does not need to input the specific system mathematical model, and can fit the complex nonlinear dynamic system well. When the PC works normally, large amounts of INS data and PC data are used as the input to the neural network to regress the incremental model of the PC heading angle. When the PC heading angle data are abnormal, the measured PC value is then compensated to provide assistance for subsequent model driving. The core of the model-driven part is the loosely coupled navigation model based on the VBCKF. Because the original measurement and the predicted measurement inevitably contain errors, and the measurement noise covariance is unknown, variational Bayesian theory is introduced to the model-driven part to estimate the virtual measurement noise covariance and improve the model’s estimation accuracy. The MEMS-INS/PC seamless integrated navigation system based on dual data and model driving can solve the problem of PC data loss in complex occlusion environments, and it can also restrain the error accumulation caused by the IMU and sensor measurement noise. The working framework for the integrated navigation system is shown in Figure 3. 

### 3.1. Design of NARX Input/Output Models

To obtain more accurate prediction results, it is necessary to select suitable training samples. At present, neural-network-assisted MEMS-INS/PC integrated navigation systems can be mainly categorized into two types. The first type establishes the relationship between the initial INS information and the output heading angle errors of the INS and the PC, i.e., $O_{INS}-\delta \varphi_{PC-INS}$, which can be expressed specifically as

(11) $$\begin{array}{ll} \delta {\hat{\varphi }}_{PC-INS} & ={\hat{\varphi }}_{PC}-{\hat{\varphi }}_{INS}\\ & =\varphi_{PC}+\delta \varphi_{PC}-\left(\varphi_{INS}+\delta \varphi_{INS}\right)\\ & ={\delta \varphi }_{PC-INS}+\delta \varphi_{PC}-\delta \varphi_{INS} \end{array}$$

where $\delta {\hat{\varphi }}_{PC-INS}$ is the heading angle error output by the two sensors; ${\hat{\varphi }}_{PC}$ and ${\hat{\varphi }}_{INS}$ represent the heading angles output by the PC and the INS, respectively; $\varphi_{PC}$ and $\varphi_{INS}$ represent the actual heading angle information of the PC and the INS, respectively; $\delta \varphi_{PC}$ and $\delta \varphi_{INS}$ represent the measurement errors of the PC and the INS, respectively; and ${\delta \varphi }_{PC-INS}$ is the actual heading angle error of the two sensors. The heading angle error $\delta \varphi_{PC-INS}$ output by this model includes the measurement errors for both sensors.

The second type establishes a relationship between the initial INS information and the PC heading angle increment, i.e., $O_{INS}-∆{\hat{\varphi }}_{PC}$, which can be expressed as

(12) $$\begin{array}{ll} ∆{\hat{\varphi }}_{PC}\left(k\right) & ={\hat{\varphi }}_{PC}\left(k\right)-{\hat{\varphi }}_{PC}\left(k-1\right)\\ & =\varphi_{PC}\left(k\right)+\delta \varphi_{PC}\left(k\right)-\left(\varphi_{PC}\left(k-1\right)+\delta \varphi_{PC}\left(k-1\right)\right)\\ & ={∆\varphi }_{PC}\left(k\right)+\delta \varphi_{PC}\left(k\right)-\delta \varphi_{PC}\left(k-1\right) \end{array}$$

where $∆{\hat{\varphi }}_{PC}(k)$ denotes the increment in the heading angle output from the PC at moment k; ${\hat{\varphi }}_{PC}(k)$ denotes the heading angle output from the PC at the moment k; $\varphi_{PC}\left(k\right)$ and $\delta \varphi_{PC}\left(k\right)$ denote the actual heading angle of the PC at the moment k and the corresponding measurement error of the PC heading angle, respectively; and ${∆\varphi }_{PC}\left(k\right)$ denotes the actual increment in the PC heading angle at the moment k. The above shows that the heading angle increment output from this model only includes the PC heading angle measurement error. The previous model includes the measurement errors of both sensors and the error $\delta \varphi_{INS}$ of INS will also accumulate with time; therefore, the input–output model selected here is the $O_{INS}-∆\varphi_{PC}$ model.

The input to the neural network is the initial information of the INS, including the specific force f and the angular velocity ω, and the output from the network is the increment in the PC heading angle $∆{\hat{\varphi }}_{PC}$, which can be expressed as

(13) $$\mathrm{Input}:\;\left[\;\begin{array}{cc} f & \omega \end{array}\right]=\left[\begin{array}{cccccc} {\;f}_{x} & f_{y} & f_{z} & \omega_{x} & \omega_{y} & \omega_{z}\; \end{array}\right]$$

(14) $$\mathrm{Output}:\left.\begin{array}{c} ∆{\hat{\varphi }}_{PC} \end{array}\right..$$

### 3.2. Nonlinear System Processing Method Based on VB

In the complex occlusion dynamic environment, time-varying noise occurs during observation of the nonlinear integrated navigation system, and the covariance matrix of the noise is usually unknown. However, the traditional Kalman filter ignores the variations and sets the measurement noise covariance at a constant value, which means that the filter is unable to track the system changes well and leads to reduced estimation accuracy. Therefore, the variational Bayesian method is introduced here to approximate the joint posterior distribution of the measurement noise covariance and estimate the measurement noise covariance.

In generalized Bayesian filtering theory, the state $x_{k}$ of the system and the covariance $R_{k}$ of the measurement noise are treated as random variables and are regarded as the parameters to be estimated. The joint prior probability density function of these two parameters at the moment k−1 can be expressed as

(15) $$p\left(x_{k},R_{k}|z_{1:k-1}\right)=\int p\left(x_{k}|x_{k-1}\right)p\left(x_{k},R_{k}|z_{1:k-1}\right)\cdot p\left(x_{k-1},R_{k-1}|z_{1:k-1}\right)dx_{k-1}dR_{k-1}$$

The joint posterior probability density function at time k is then

(16) $$\begin{array}{ll} p\left(x_{k},R_{k}|z_{1:k}\right) & =\frac{p\left(x_{k},R_{k},z_{1:k}\right)}{p\left(z_{1:k}\right)}\\ & =\frac{p\left(z_{k},z_{1:k-1}|x_{k},R_{k}\right)p\left(x_{k},R_{k}\right)}{p\left(z_{k},z_{1:k-1}\right)}\\ & =\frac{p\left(z_{k}|x_{k},R_{x}\right)p\left(x_{k},R_{k}|z_{1:k-1}\right)}{p\left(z_{k}|z_{1:k-1}\right)} \end{array}$$

The equations above can be used as a summary of the prediction and updating steps of the generalized Bayesian filtering method. However, in practical applications, because (15) and (16) contain complex integral operations, it is difficult to obtain analytical solutions using this method. Therefore, variational Bayesian theory [20] is introduced here to obtain approximate solutions, and (15) and (16) are approximated as the products of the Gaussian distribution and the inverse gamma distribution:

(17) $$p\left(x_{k},R_{k}|z_{1:k-1}\right)\approx N\left({\hat{x}}_{k|k-1},P_{k|k-1}\right)\cdot \prod_{i=1}^{b}Inv\text{-}Gamma\left(\sigma_{k,i}^{2}|\lambda_{k|k-1,i},\mu_{k|k-1,i}\right)$$

(18) $$p\left(x_{k},R_{k}|z_{1:k}\right)\approx N\left({\hat{x}}_{k},P_{k}\right)\cdot \prod_{i=1}^{b}Inv\text{-}Gamma\left(\sigma_{k,i}^{2}|\lambda_{k,i},\mu_{k,i}\right)$$

where ${\hat{x}}_{k}$ is the state estimation at time *k*; $P_{k}$ is the estimated covariance at time *k*; *b* is the dimension of the measurement variance; $\sigma_{k,i}^{2}$ is the unknown variance of the Gaussian distribution of the measurement noise; and Inv-Gamma(⋅) represents the inverse gamma distribution. $\lambda_{k|k-1,i}$ and $\mu_{k|k-1,i}$ are the inverse gamma distribution parameters and can be expressed as

(19) $$\lambda_{k|k-1,i}=\rho_{i}\lambda_{k-1,i},\;\mu_{k|k-1,i}=\rho_{i}\mu_{k-1,i},$$

where $\rho_{i}$ is the variational attenuation coefficient taking values in the interval (0,1].

Ultimately, the measurement noise covariance can be expressed as

(20) $$R_{k}=diag\left(\mu_{k,1}/\lambda_{k,1},\cdots ,\mu_{k,b}/\lambda_{k,b}\right).$$

The equations above represent the derivation of the VB algorithm, but this algorithm is only applicable to linear systems. For the nonlinear navigation model given in Section 2.1, the CKF algorithm must be introduced to solve the nonlinear problem. In this work, the VB algorithm is combined with the CKF algorithm to obtain the VBCKF based on the VB, and its specific filtering process is given as follows:

Step 1: Initialization

(21) $${\hat{x}}_{0}=E\left(x_{0}\right),$$

(22) $$P_{0}=E\left[\left(x_{0}-{\hat{x}}_{0}\right){\left(x_{0}-{\hat{x}}_{0}\right)}^{T}\right].$$

Step 2: Time update

Calculate the cubature point at *k* − 1:

(23) $$P_{k-1}=S_{k-1}S_{k-1}^{T}$$

(24) $$x_{k-1,i}=S_{k-1}\xi_{i}+{\hat{x}}_{k-1}$$

where $S_{k-1}$ denotes the square root of the covariance of the state’s prior distribution, and $\xi_{i}$ is the cubature point, which is defined as

(25) $$\xi_{i}=\sqrt{\frac{m}{2}}I_{i},i=1,2,\cdots ,m$$

where m=2n is the number of cubature points, *n* is the dimension of the state vector, and $I_{i}$ can be expressed as

(26) $$I_{i}=\left\{\begin{array}{cccccccc} 1 & 0 & \cdots & 0 & -1 & 0 & \cdots & 0\\ 0 & 1 & \cdots & 0 & 0 & -1 & \cdots & 0\\ \vdots & \vdots & \cdots & \vdots & \vdots & \vdots & \cdots & \vdots \\ 0 & 0 & \cdots & 1 & 0 & 0 & \cdots & -1 \end{array}\right\}$$

Propagate the cubature point:

(27) $$x_{k|k-1,i}=f\left(x_{k-1,i}\right)$$

Predict the state:

(28) $${\hat{x}}_{k|k-1}=\frac{1}{m}\sum_{i=1}^{m}\;x_{k|k-1,i}$$

(29) $$P_{k|k-1}=\frac{1}{m}\sum_{i=1}^{m}\;x_{k|k-1,i}x_{k|k-1,i}^{T}-{\hat{x}}_{k|k-1}{\hat{x}}_{k|k-1}^{T}+Q_{k}$$

(30) $$\lambda_{k|k-1,i}=\rho_{i}\lambda_{k-1,i}\;\;\;\;\;i=1,2,\ldots ,b$$

(31) $$\mu_{k|k-1,i}=\rho_{i}\mu_{k-1,i}\;\;\;\;\;i=1,2,\ldots ,b$$

Step 3: Measurement update

Calculate and propagate the volume points:

(32) $$P_{k|k-1}=S_{k|k-1}S_{k|k-1}^{T}$$

(33) $$X_{k|k-1,i}=S_{k|k-1}\xi_{i}+{\hat{x}}_{k|k-1}$$

(34) $$z_{k|k-1,i}=h\left(X_{k|k-1,i}\right)$$

Calculate the predicted measurements:

(35) $${\hat{z}}_{k|k-1}=\frac{1}{m}\sum_{i=1}^{m}\;z_{k|k-1,i}$$

Calculate the cross covariance:

(36) $$P_{xz}=\frac{1}{m}\sum_{i=1}^{m}\;X_{k|k-1,i}z_{k|k-1,i}^{T}-{\hat{x}}_{k|k-1}\;{\hat{z}}_{k|k-1}^{T}$$

Update the parameters of the inverse gamma distribution:

(37) $$\lambda_{k,i}=1/2+\lambda_{k|k-1,i},\mu_{k,i}=\mu_{k|k-1,i}$$

Perform N iterations to calculate the covariance ${\hat{R}}_{k}^{n}$ of the measurement noise:

(38) $${\hat{R}}_{k}^{n}=diag(\mu_{k,1}^{n}/\lambda_{k,1}^{n},\ldots ,\mu_{k,d}^{n}/\lambda_{k,d}^{n})$$

Calculate the self-covariance:

(39) $$P_{zz}^{n+1}=\frac{1}{m}\sum_{i=1}^{m}\;(z_{k|k-1,i}-{\hat{z}}_{k|k-1}){\left(z_{k|k-1,i}-{\hat{z}}_{k|k-1}\right)}^{T}+{\hat{R}}_{k}^{n}$$

Update the filter gain, the state, and the covariance:

(40) $$K_{k}^{n+1}=P_{xz}/P_{zz}^{n+1}$$

(41) $${\hat{x}}_{k}^{n+1}={\hat{x}}_{k/k-1}+K_{k}^{n+1}\left(z_{k}-{\hat{z}}_{k/k-1}\right)$$

(42) $$P_{k}^{n+1}=P_{k|k-1}-K_{k}^{n+1}P_{zz}^{n+1}({K_{k}^{n+1})}^{T}$$

$Conducta\;\mu_{k,i}$ posterior update:

(43) $$z_{k,i}^{n+1}=h\left({\hat{x}}_{k}^{n+1}\right)$$

(44) $$\mu_{k,i}^{n+1}=\mu_{k|k-1,i}+\frac{1}{2m}\sum_{i=1}^{m}\;\left(z_{k}-z_{k,i}^{n+1}\right){\left(z_{k}-z_{k,i}^{n+1}\right)}^{T}$$

Stop the iteration when *n = N*, and let $\mu_{k,i}=\mu_{k,i}^{N}$*,* ${\hat{x}}_{k}={\hat{x}}_{k}^{N}$, and $P_{k}=P_{k}^{N}$. To ensure the accuracy and speed of the algorithm, the number of iterations was set at N = 3 in this work.

At this point, the complete VBCKF filtering process is over. In the measurement update step, the improved filter uses the VB method to estimate the covariance of the measurement noise iteratively, and then it updates the system state.

### 3.3. MEMS-INS/PC Loosely Coupled Navigation Method Based on NARX-VBCKF

When the PC signal is available, as shown in Figure 3a, the integrated navigation system is in its training mode. The inputs to the NARX include the specific force f and the angular velocity ω from the INS, as shown in (13). The incremental heading angles $∆{\hat{\varphi }}_{PC}$ calculated from the PC heading angles collected at different moments in time are used as the network outputs, as shown in (14). The GNBR training algorithm is used to determine the relationship between the heading angle increment $∆{\hat{\varphi }}_{PC}$, the specific force f, and the angular velocity ω. Simultaneously, the INS information and the PC information are input into the loosely coupled navigation model based on the VBCKF for optimal estimation to obtain the heading angle error of the INS; the heading information is then corrected to obtain the optimal heading angle.

In the case of lost or inaccurate PC signals, as shown in Figure 3b, the integrated navigation system shifts into its predictive mode. At this point, the specific force f and the angular velocity ω of the INS are still available, and using this information as the inputs to the trained neural network allows the neural network to predict the angular increment in the PC heading $∆{\hat{\varphi }}_{PC}$. The PC heading angle at the current moment can be obtained by accumulating the heading angle increments from the time that the predictions started. The new PC heading angle can replace the original, inaccurate PC heading angle information that was input into the VBCKF-based loosely coupled navigation model to estimate the INS heading angle error, and the optimal heading angle can then be obtained by correcting the INS heading angle. This improves the accuracy of the integrated navigation system in case of PC signal loss or inaccuracy.

## 4. Field Test and Analysis

**Figure 4:**
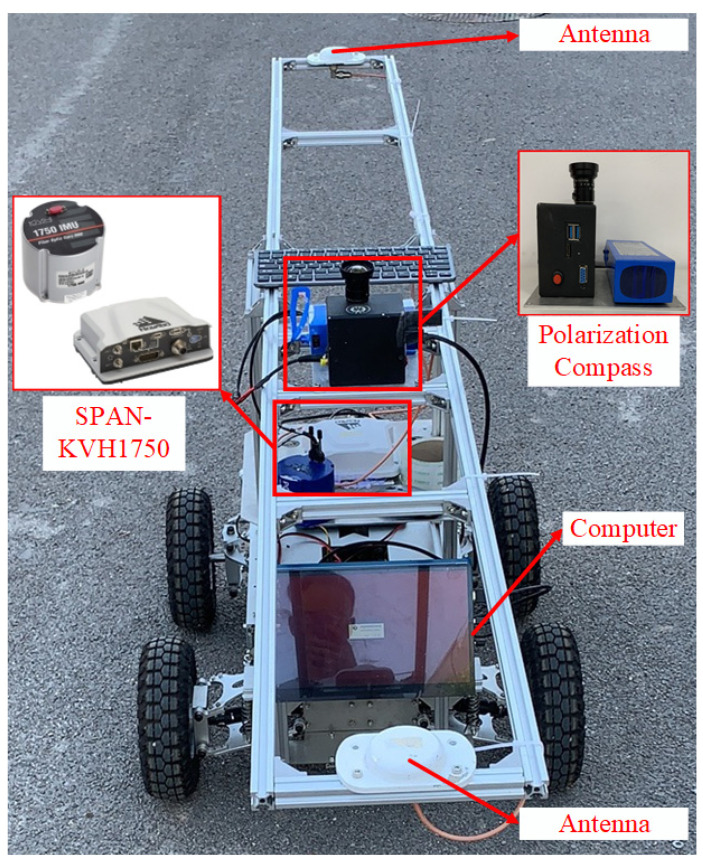
Field testing equipment.

To verify the proposed data- and model-driven MEMS-INS/PC seamless navigation method, field testing was conducted on an in-house-built UV platform using an INS and an in-house-made PC. The MEMS-INS/PC integrated navigation system and the experimental setup are shown in Figure 4. The system and sensor parameters are given in Table 1. The integrated navigation system consists of an INS, a PC, and a development board integrated and packaged in a carbon fiber enclosure. The system can collect the raw information from gyroscopes, accelerometers, and the PC. The reference navigation data come from a high-precision navigation system (Model: SPAN-KVH1750), and the computer is mainly used to receive and process the data. To evaluate the superiority of the proposed algorithm, the dynamic experiment was performed using the UV, and the following nine methods were compared:

(1) “Reference” indicates the output from the reference navigation system.
(2) “Pure INS” indicates the INS output alone.
(3) “PC” indicates the PC output.
(4) “BP” denotes compensation using the BPNN.
(5) “RBF” denotes compensation using the RBF.
(6) “NARX” denotes compensation using the NARX.
(7) “NARX-EKF” represents the extended Kalman filter (EKF) algorithm based on the NARX.
(8) “NARX-CKF” represents the CKF algorithm based on the NARX.
(9) “NARX-VBCKF” represents the proposed algorithm.

**Table 1:** Sensor details.

Sensor	Parameter	Value
INS	Heading angle accuracy	0.2°/min
PC	Frame rate	22FPS
SPAN-KVH1750	Heading angle accuracy	0.035°

During testing, to evaluate the effectiveness of the proposed algorithm in complex occluded environments and large-scale maneuver scenarios, we set up two complete occlusions and one incomplete occlusion during the UV steering process. These three cases are described as follows:

Case 1: During the 90–110 s steering period, the lens cover was used to cover the PC completely; 

Case 2: During the 135–150 s steering period, time-varying measurement noise covariance was generated through the shelter of leaves; 

Case 3: During the 180–195 s steering period, the lens cover was used to cover the PC completely.

### 4.1. Comparison of Different Neural Networks

**Figure 5:**
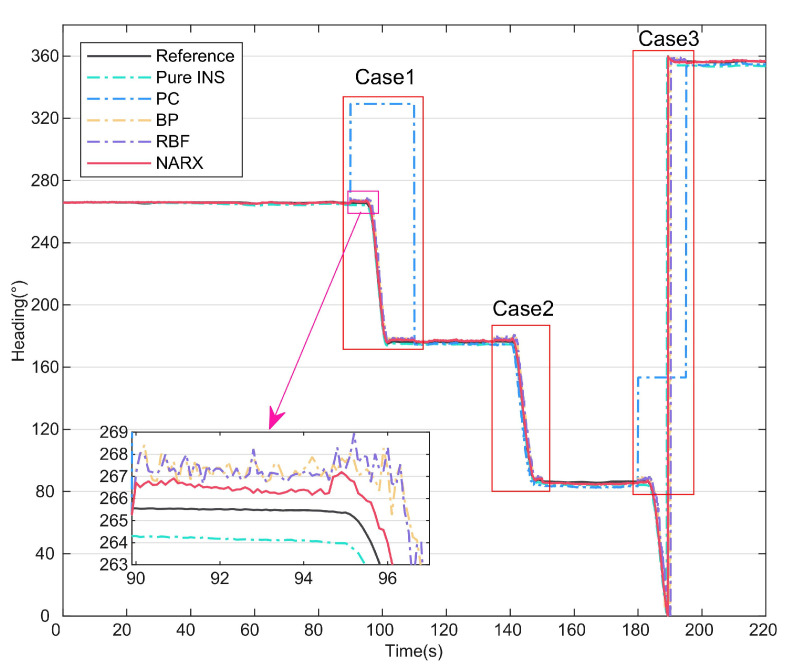
Heading angles compensated by different neural networks.

In this part of the study, to verify the effectiveness of the NARX data-driven method during PC information loss or anomalies, the proposed method was compared with other traditional neural network algorithms, including the BPNN and RBF, and the predictive compensation effects of the different neural networks were then analyzed. The test was conducted at 8:00 on 30 May 2023 at No. 3, Xueyuan Road (112.45° E, 38.02° N), Taiyuan, China. The heading of the UV is shown in Figure 5, and the periods of the three test cases are indicated. In cases 1 and 3, the PC output had a fixed value because the polarization sensor was completely obscured, and in case 2, a small variation occurred in the PC output because of measurement noise. During the complete travel process of the UV, the INS data and the PC data collected during the 0–90 s period were used as inputs to the neural network to obtain the network model, and the PC heading angle was then predicted online using the network model in cases 1–3. The magnified portion of Figure 5 shows that the prediction of the NARX neural network was better than that of the other two methods.

**Figure 6:**
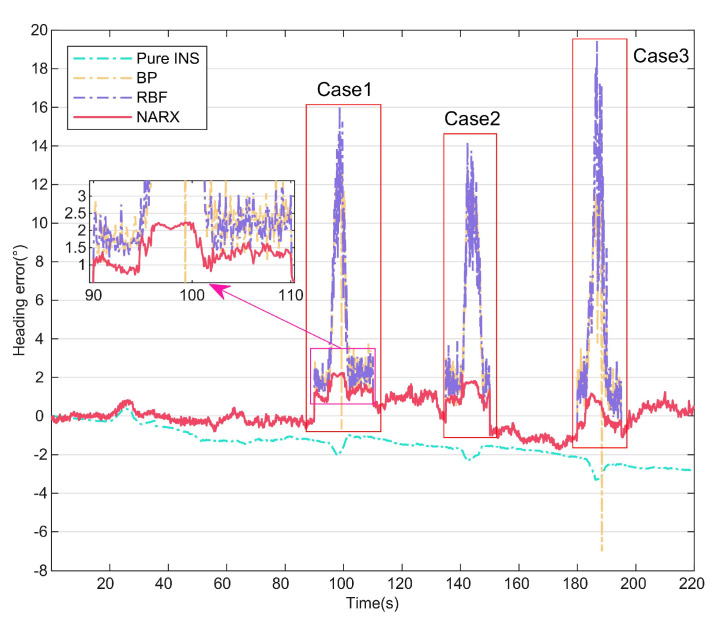
Heading angle errors compensated by different neural networks.

To illustrate the superiority of the NARX more intuitively, the error curves for Methods 2, 4, 5, and 6 are shown in Figure 6, and the statistical properties of the three neural network methods are summarized in Table 2. As shown in Figure 6, the error accumulates over time in the pure INS mode. When the NARX is used to perform predictive compensation, the heading angle error is suppressed to some extent. The enlarged portion of Figure 6 shows that the maximum error of the NARX-assisted navigation method is no more than 2.5°, which is related to the NARX’s improved dynamic adaptability based on time series analysis and prediction. In contrast, the other two neural-network-assisted navigation methods can have maximum errors of more than 12° because of their inability to capture the time dependencies in the data. Table 2 shows that the root-mean-square (RMS) error of the NARX is 70.61% lower than that of the BPNN and 72.48% lower than that of the RBF; these results prove that the NARX-assisted navigation method improves the heading angle compensation accuracy effectively when the PC information is lost or abnormal.

### 4.2. Comparison of Different Integrated Methods

**Figure 7:**
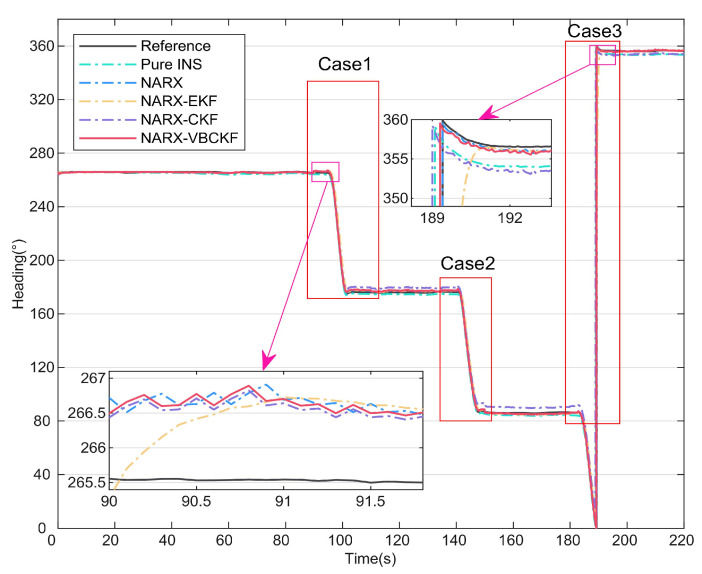
Heading angles for different integrated methods.

**Figure 8:**
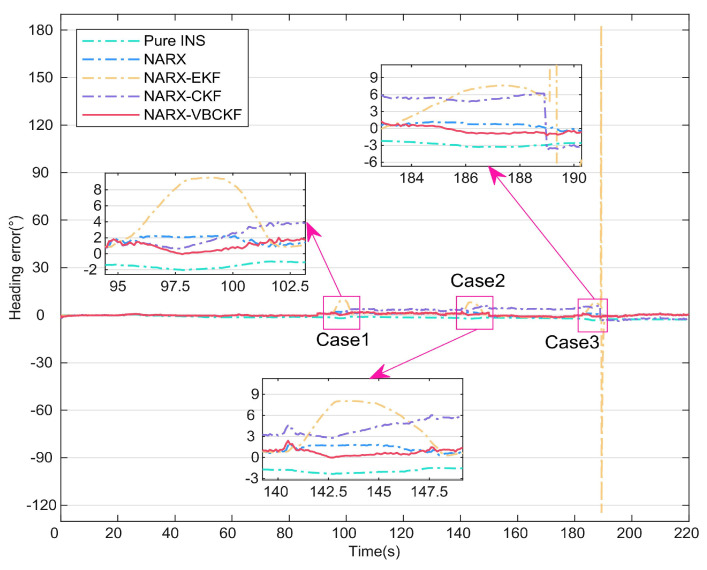
Heading angle errors for different integrated methods.

In this section, to provide further validation of the effectiveness of the model-driven approach, different filtering model algorithms are compared based on the data-driven approach presented in the previous section. The heading angles for the six methods are shown in Figure 7. The errors of these different methods are then shown in Figure 8. Table 3 lists the statistical properties of the heading angle errors for the different methods. The two enlarged sections of Figure 7 show that the NARX-EKF filtering method suffers from hysteresis, leading to large fluctuations in the error curve, and is thus unacceptable. As shown in the three enlarged sections of Figure 8, after 90 s, the accuracy of the NARX-EKF and NARX-CKF methods decreases significantly because of the effects of measurement noise. In contrast, the NARX-VBCKF method can always maintain high filtering accuracy because of the introduction of the VB approach to suppress the effects of time-varying measurement noise on the combined model. Table 3 shows that when compared with the NARX-EKF and NARX-CKF methods, the NARX-VBCKF heading angle errors are reduced by 87.96% and 72.53%, respectively. These results indicate that the NARX-VBCKF method proposed in this article offers the best filtering accuracy, and all its measurement indexes are comparatively superior.

In summary, among the integrated methods described above, only the proposed dual data- and model-driven MEMS-INS/PC seamless navigation method can satisfy the navigation needs of UVs in both complex occlusion environments and large-scale maneuvering processes. In addition, the robustness and accuracy of the proposed NARX-VBCKF method are better than those of the other integrated methods described above.

## 5. Conclusions

In this article, a new seamless MEMS-INS/PC navigation method based on a dual data- and model-driven approach has been proposed to reduce heading error accumulation in IoT applications, such as autonomous driving. In the data-driven part, a NARX based on the GNBR training algorithm is used to deal with the nonlinear relationship between the INS information and the PC heading angle increment; this relationship effectively captures the time dependence in the data and simultaneously ensures that the most important input variables are selected without overfitting the model. In the model-driven part, a nonlinear MEMS-INS/PC loosely coupled integrated navigation model is developed to suppress the effects of time-varying measurement noise on the integrated navigation system by introducing the VB method, while the CKF method is used to deal with the nonlinearities in the model. Field test results show that the proposed method improves the estimation accuracy and the robustness of the integrated navigation system in cases of anomalous measurements and unknown measurement noise covariance when compared with the traditional purely model-driven integrated navigation method. Future work will consider the addition of a geomagnetic compass to the integrated navigation system to enable pitch and roll angle compensation.

## Figures and Tables

**Table 2:** RMS, MEAN, MAX, and MIN heading angle errors for the three neural network methods (unit: °).

Method	RMS	Mean	Max	Min
BP	2.79	0.91	12.96	−7.01
RBF	2.98	0.96	19.43	−1.74
NARX	**0.82**	**0.15**	**2.24**	**−1.73**

**Table 3:** RMS, mean, max., and min. heading angle errors for the three integrated methods (unit: °).

Method	RMS	Mean	Max.	Min.
NARX-EKF	6.23	0.49	183.2	−124.7
NARX-CKF	2.73	1.20	6.29	−3.87
NARX-VBCKF	**0.75**	**0.13**	**2.38**	**−1.52**

## Data Availability

The datasets presented in this article are not readily available because the data are part of an on-going study.
